# Unraveling the Complexity of Multicentric Gliomas: Insights Into Chronicity and Genetic Aberrations

**DOI:** 10.7759/cureus.37284

**Published:** 2023-04-08

**Authors:** Ali Msheik, Youssef Fares, Maarouf Hamoud, Rami Atat

**Affiliations:** 1 Neurosurgery, Al Zahraa Hospital University Medical Center, Beirut, LBN; 2 Neurology Division, Faculty of Medicine, Lebanese University, Al Zahraa Hospital University Medical Center, Beirut, LBN

**Keywords:** multicentric gliomas, primary brain tumors, mutlifocal gliomas, astrocytoma, mutations and polymorphisms studies

## Abstract

Gliomas are among the most common primary tumors of the brain. Discrimination among tumors of more than one focus has segregated the latter into two groups: multifocal gliomas and multicentric gliomas (MCGs). In this case series, outcomes among three patients are described and discussed in light of the findings present in the literature. Ideally, it is crucial to consider genetic testing for categorizing each tumor. This can help determine the original genetic mutations of MCGs and allow to establish necessary screening testing for early detection. We present the cases of three patients diagnosed with cranial gliomas. The first case showed two synchronous gliomas at different loci in the right hemisphere. The second patient showed synchronous lesions on cranial magnetic resonance imaging in each hemisphere. The third case was of a patient with metachronous lesions appearing at different times with similar radiological findings at different loci of the same hemisphere. Discrimination among multifocal and multicentric gliomas requires genetic workup because radiological and temporal findings may fail to allow adequate discrimination.

## Introduction

Brain tumors are a complex and heterogeneous group of neoplasms. Over 120 different types of brain tumors have been identified based on the specific brain tissue affected [1]. Gliomas are the most common primary malignant brain tumors, accounting for approximately 80.8% of reported cases [2]. Unfortunately, the prognosis for patients with glioblastoma, the most aggressive form of glioma, is generally poor, with a median survival time of approximately 16-21 months and as short as four months as reported [3,4].

Traditionally, glioblastomas were thought to occur uniformly, but recent research has suggested that this notion may not be entirely accurate. It is now believed that gliomas are a family of diverse progenitor cells that failed to differentiate due to genetic defects in stem cells, leading to the formation of a specific population of defective hyperproliferative cells that form the tumor center, or multicentric glioma (MCG) [5,6]. In contrast, multifocal gliomas (MFGs) represent the occurrence of multiple tumor foci with distinct associations and consistent genetic deviations [7-9].

The concept of glioblastoma recurrence occurs in the majority of patients in situ due to incompletely resected tumors and is different in the remaining minority due to metastases along white matter pathways and cerebrospinal fluid (CSF) spread occurring locally [7,8]. This is the concept of the theory behind the formation of synchronous or metachronous foci, i.e. multifocal gliomas. Tumors that recur without evidence of metastasis are called metachronous multicentric gliomas. The co-occurrence of glioma without evidence of connection explains isochronous MCGs [9]. Genetic differences between lesions that cannot be explained by metastasis theory promote this discrimination.

One or more critical genetic mutations cause cell division defects, and MCG formation is determined by multiple directional migrations in which each cell lineage undergoes a series of genetic mutations [10]. This indicates that genetic mutations shared in MCG centers may be the earliest genetic abnormalities, enabling screening tools in high-risk populations [10,11].

Here, we have described cases of three patients diagnosed with malignant gliomas located in different regions of the brain. Further discussion is provided concerning the literature results.

## Case presentation

Case 1

**Figure 1:**
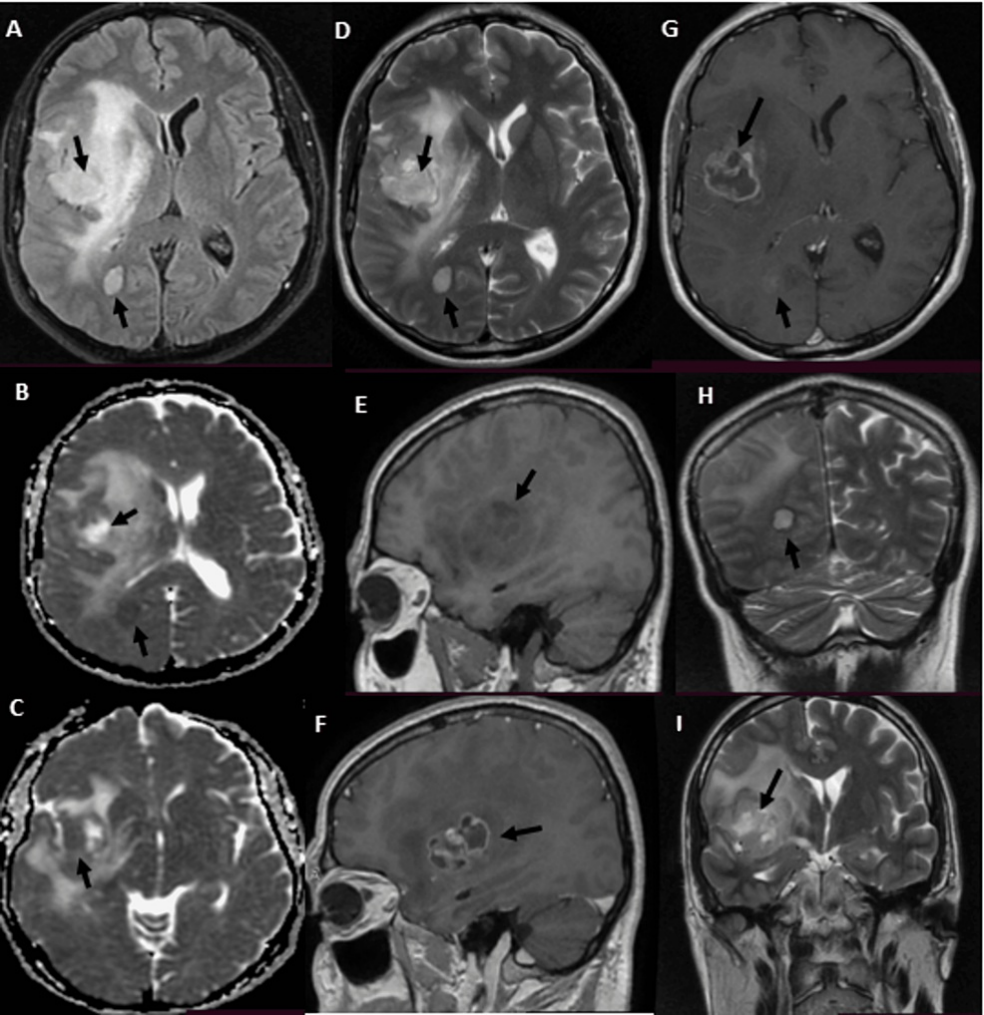
Cranial MRI showing the two lesions, with different contrast uptakes, different FLAIR sequence intensities and different diffuse sequence intensities (A) T1 sequence axial view without contrast; (B, C) DWI axial view; (D) T2 axial view; (E) T1 sequence sagittal view without contrast; (F) T1 sequence sagittal view with contrast; (G) T1 sequence axial view with contrast; (H, I) T2 sequence coronal view FLAIR, fluid-attenuated inversion recovery; DWI, diffusion-weighted; MRI, magnetic resonance imaging Image courtesy: Al Zahraa Hospital University Medical Center (ZHUMC) Radiology Department

In July 2019, a sudden onset of headache and left-sided weakness associated with a left Babinski sign was observed in a 48-year-old male patient. A brain MRI scan showed a hyper-intense signal on T2 fluid-attenuated inversion recovery (FLAIR) and diffusion-weighted imaging (DWI) sequences and low apparent diffusion coefficient (ADC) signal in a right temporo-frontal mass with a hypo-intense signal on the T1 sequences (Figure 1). Diffuse edema was also observed around the mass. Another mass was detected in the right occipital region, which had similar signal findings in the aforementioned sequences, but showed weak contrast uptake. The patient was administered 500 mg valproic acid orally (per os) twice daily and a dexamethasone loading dose of 10 and 8 mg twice daily intravenously. A frontotemporal craniotomy was performed during the same admission to excise the right-sided frontotemporal tumor. A stereotactic biopsy was done for the right-sided occipital region during the same procedure. A pathological examination of both tumors showed grade IV glioblastoma multiforme (GBM) astrocytoma.

Case 2

**Figure 2:**
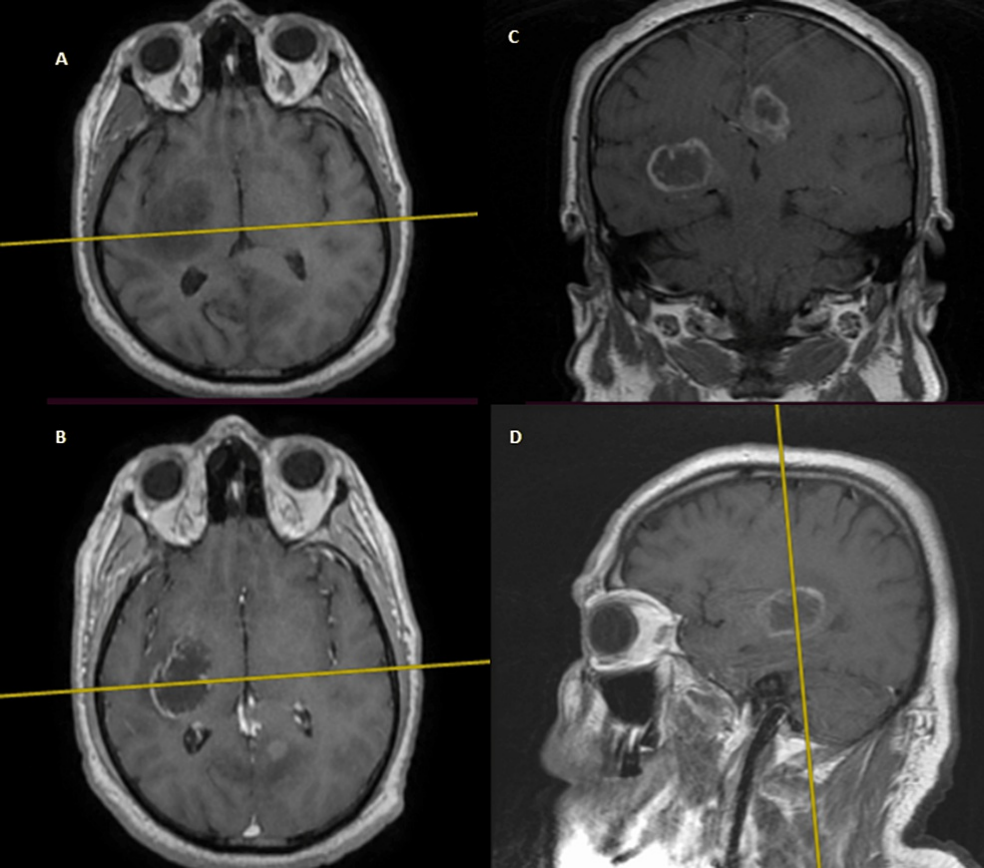
Cranial MRI showing the first lesion (insular right-sided, all T1 sequences) (A) Axial cut without contrast; (B) axial cut with contrast; (C) coronal cut with contrast; (D) sagittal cut with contrast Image courtesy: Al Zahraa Hospital University Medical Center (ZHUMC) Radiology Department

**Figure 3:**
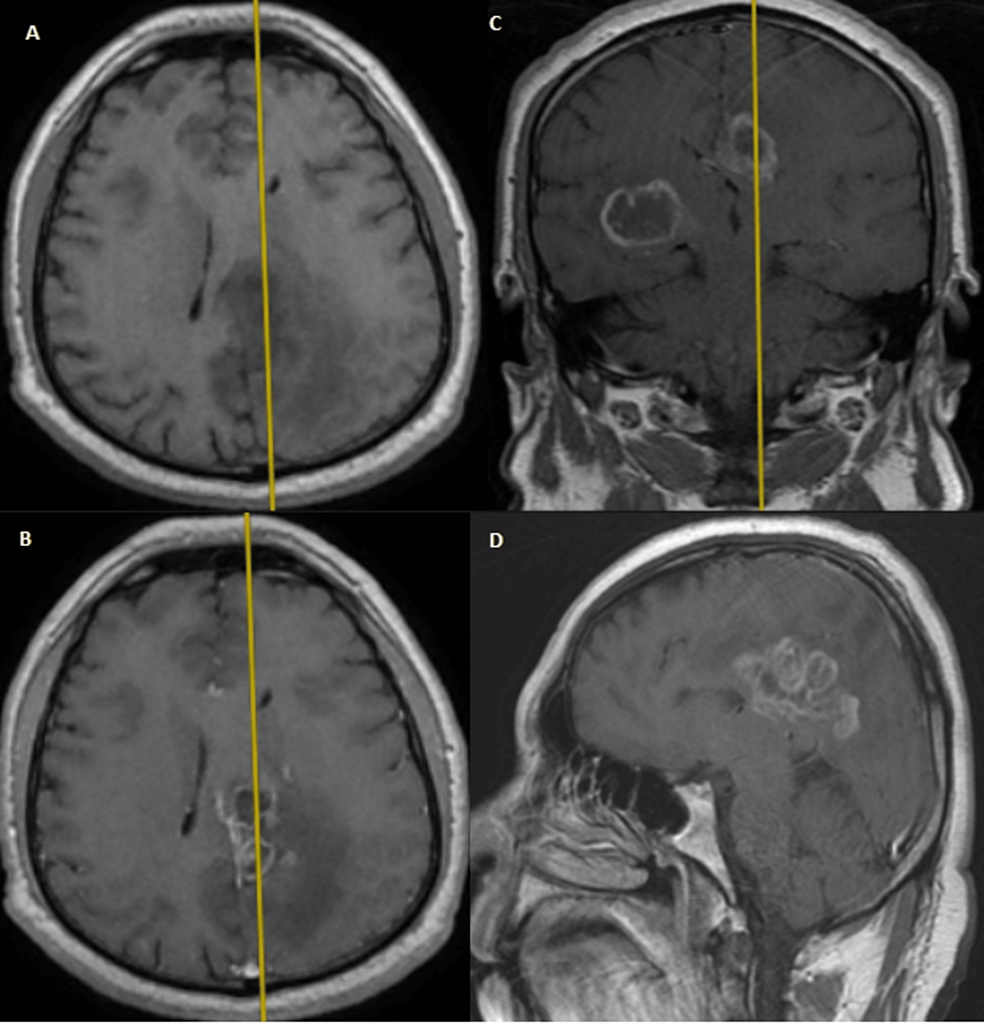
Cranial MRI showing the second lesion (parietal left-sided, all T1 sequences) (A) Axial cut without contrast; (B) axial cut with contrast; (C) coronal cut with contrast; (D) sagittal cut with contrast Image courtesy: Al Zahraa Hospital University Medical Center (ZHUMC) Radiology Department

In November 2019, a 56-year-old male patient was admitted with symptoms of intractable vomiting, blurred vision, left-sided weakness, and headache. Brain MRI scans revealed two lesions, with both exhibiting similar hyper-intense signal on T2, T2 FLAIR, and DWI signal sequences, a hypo-intense signal on T1 sequences, and a similar contrast uptake (Figures 2, 3). The first lesion was located in the left frontoparietal region, while the second lesion was found in the right temporal region. The patient underwent right-sided craniotomy for the excision of the right-sided lesion and left-sided craniectomy for the biopsy of the left-sided lesion. Both pathologies were found to be high-grade IV astrocytoma. Following the procedure, the patient was discharged without complications and placed on valproic acid 500 mg per os twice daily and a course of tapered dexamethasone.

Case 3

**Figure 4:**
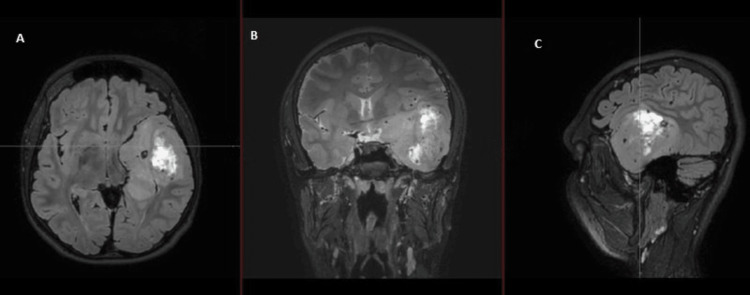
Cranial MRI showing the first lesion in the left temporal area (T1 sequences without contrast, before surgery) (A) Axial view; (B) coronal view; (C) sagittal view Image courtesy: Al Zahraa Hospital University Medical Center (ZHUMC) Radiology Department

**Figure 5:**
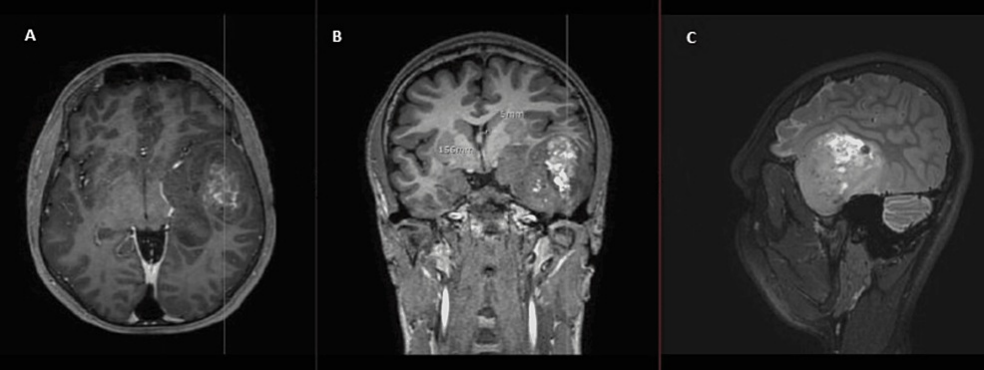
Cranial MRI showing the first lesion in the left temporal area (T1 sequences with contrast, before surgery) (A) Axial view; (B) coronal view; (C) sagittal view The distance from the midline to the lateral border of the tumor is 156 mm; the distance from the midline to the medial border is 5 mm, in the coronal section. Image courtesy: Al Zahraa Hospital University Medical Center (ZHUMC) Radiology Department

**Figure 6:**
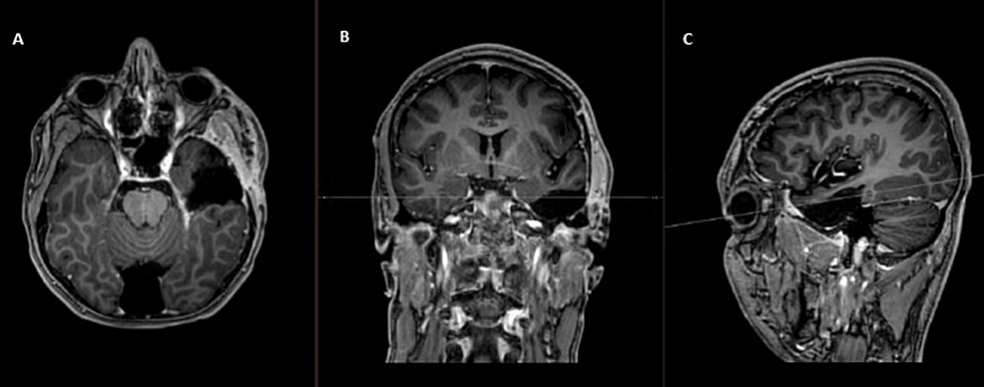
Cranial MRI one month after left temporal lobectomy (T1 sequences without contrast) (A) Axial view; (B) coronal view; (C) sagittal view Image courtesy: Al Zahraa Hospital University Medical Center (ZHUMC) Radiology Department

**Figure 7:**
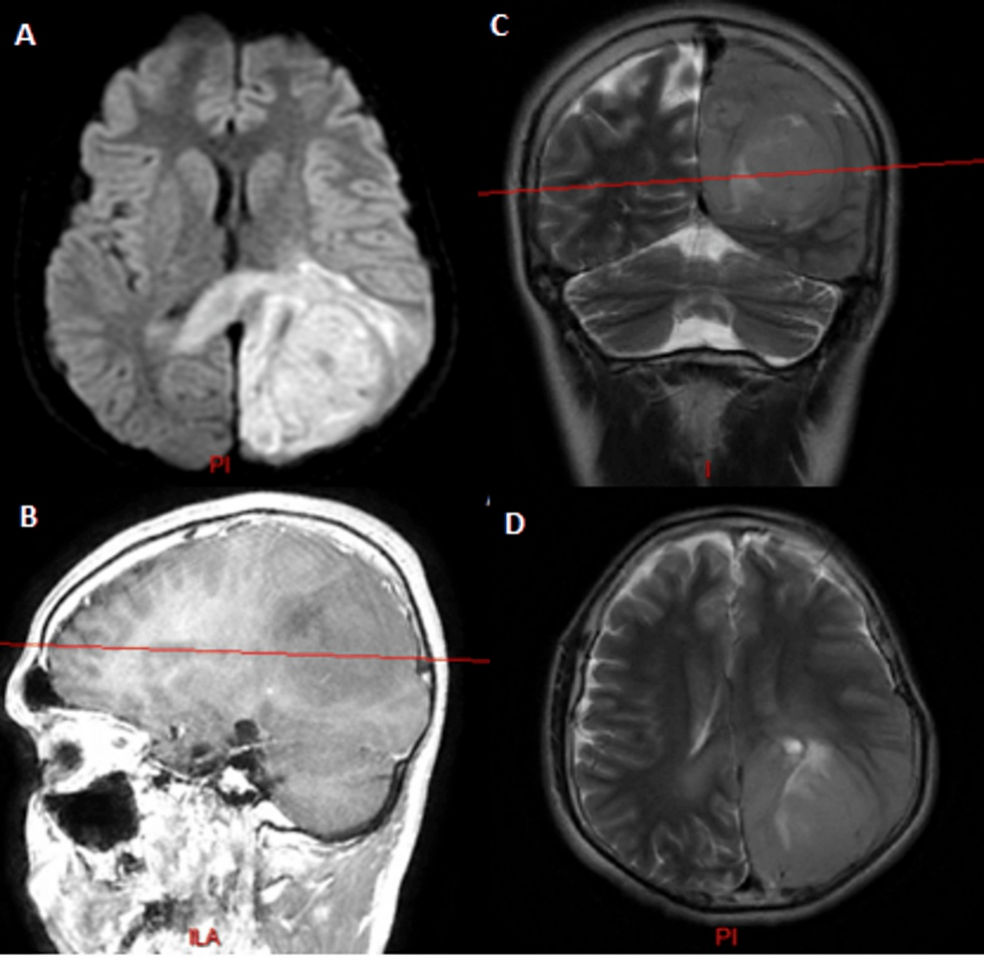
Cranial MRI showing the second lesion developed eight months after left temporal lobectomy (A) Diffusion-weighted image; (B) T1 sequence sagittal view with contrast; (C) T2 sequence coronal view; (D) T2 sequence axial view PI, postero-inferior; I, inferior; ILA, infero-lateral aspect Image courtesy: Al Zahraa Hospital University Medical Center (ZHUMC) Radiology Department

A 16-year-old male patient presented in September 2021 with symptoms of headache, seizures, and right-sided motor weakness. A cranial MRI scan showed a hyper-intense T1 FLAIR sequence signal and moderate contrast uptake in a left-sided temporal lesion. During the same admission, the patient underwent a craniotomy with left temporal lobectomy, and the pathology results revealed a pediatric high-grade astrocytoma. The patient was readmitted in May 2022 due to seizures and recurrent right-sided weakness, and a new lesion was detected in the left occipital lobe. The patient was treated with dexamethasone and anti-epileptics, but intratumoral bleeding occurred, leading to brain herniation and the patient's death. (Figures 4-7 depict MRI scans and surgical procedure.)

## Discussion

The first patient in this case series showed a large, right frontotemporal lesion that behaved differently compared to the smaller right occipital lesion, which showed no contrast uptake on T1 sequence images. Depending on imaging, anatomic criteria, and pathology results, this patient most probably had an MCG. Although these lesions are visible at the same time, metachronous formation and development could be considered. The second patient showed two synchronous lesions with different anatomic locations each in a hemisphere. As a connection through CSF or white matter pathways could be possible, an MFG could not be ruled out. The third patient showed two metachronous lesions with similar necrotic behavior, yet a different anatomic location. Attribution of the second lesion to metastasis of the first one hindered the confident diagnosis of an MCG. Genetic testing could provide evidence for the second and third cases and ultimately provide a definitive diagnosis [10].

These cases are remarkable because they present unique and rare cases of multiple cerebral gliomas, including multicentric gliomas and multifocal gliomas. These cases are of particular interest to the medical community due to the rarity and complexity of these tumors, which make diagnosis and treatment challenging. The first case demonstrated the difference in behavior between a large right frontotemporal lesion and a smaller right occipital lesion, making it difficult to diagnose an MCG. The second case showed two synchronous lesions with different anatomic locations, leading to uncertainty about whether it was an MCG or an MFG. The third case presented two metachronous lesions with similar necrotic behavior but different locations, making it difficult to attribute the second lesion to the metastasis of the first one. These cases highlight the importance of distinguishing between MCGs and MFGs, utilizing imaging and genetic testing, and considering both anatomic and genetic clues in the diagnosis. These cases will contribute to the understanding of the clinical presentation, imaging features, and molecular characteristics of these rare and complex tumors, which can ultimately improve the management and outcomes of patients with similar conditions.

Genetic testing, imaging, and anatomic positioning of lesions help discriminate the MCGs from MFGs [11,12]. The utilization of FLAIR sequences on MRI discerns the disjointness of lesions as a characteristic of MCGs. Failure to provide evidence of anatomic connections through white matter pathways and CSF or metastasis advocates the diagnosis of MCGs over MFGs [10]. Currently, the focus is on the genetic basis that allows lesions to be organized into a genetic tree, with each lesion occupying a specific position within it. Genetic analysis and mutation determination are of several benefits. First, MFGs can be less mistakenly diagnosed as MCGs due to anatomic considerations because of genetic clues of disjointness. When there is a contradiction between genetic analysis and anatomic correlation, many researchers favor the genetic basis over the anatomic findings. The relatively low incidence of MCGs, reported as low as 2%, would then increase [10,13,14]. Of note, the low incidence of MCGs can be attributed to other reasons including the neglect of small lesions on MRI, and loss of follow-up of patients with metachronous MCGs.

## Conclusions

Evidence of multiple foci of a brain tumor can be suggestive of the process of either multiple centers of neoplasms or connected foci with similar genetic mutations, hence, the terminology of MCGs and MFGs. The discrimination of each type has depended on the literature on radiological evidence and assessment of chronicity among tumor foci. However, consideration of genetic testing can not only allow for segregation of tumors into either category but also lead to the establishment of screening tools for genetic mutations that can be helpful in the early detection of these high-mortality gliomas.
